# Self-directed arm-crank exercise to improve volitional control of the trunk in patients with subacute spinal cord injury: a multicentre, parallel-group, randomised controlled trial protocol

**DOI:** 10.1136/bmjopen-2024-092226

**Published:** 2025-08-21

**Authors:** Maria del Rocio Hidalgo Mas, Joshua Kearney, Vicki Middleton, Chuang-Yuan Chiu, Joan L Duda, Tom E Nightingale, Eduardo Martinez-Valdes, Zubair Ahmed, Shin-Yi Chiou

**Affiliations:** 1 School of Sport, Exercise and Rehabilitation Sciences, University of Birmingham, Birmingham, UK; 2 Sheffield Teaching Hospitals NHS Foundation Trust, Sheffield, UK; 3 Centre for Sports Engineering Research, Sheffield Hallam University, Sheffield, UK; 4 Neuroscience and Ophthalmology, Institute of Inflammation and Ageing, University of Birmingham, Birmingham, UK; 5 University Hospitals Birmingham NHS Foundation Trust, Edgbaston, Birmingham, UK

**Keywords:** Neurophysiology, Physical Therapy Modalities, Clinical Trial, Posture, Quality of Life

## Abstract

**ABSTRACT:**

**Introduction:**

A spinal cord injury (SCI) disrupts synaptic connections between the corticospinal tract and motor neurons, impairing muscle control below the injury site. Many individuals with an SCI have impaired trunk control, affecting the performance of activities of daily living and quality of life. Work has shown improvements in trunk control after home-based, unsupervised arm-crank exercise training (ACET) in people with chronic motor-incomplete SCI. However, no studies have examined ACET’s impact on trunk control in individuals with subacute SCI. This study aims to investigate ACET’s effects on trunk control in adults with subacute incomplete SCI, and its mechanisms, and its long-term benefits on neuropathic pain, psychological well-being, physical activity levels and health-related quality of life.

**Methods and analysis:**

This multicentre, parallel-group, randomised controlled trial will evaluate self-directed ACET in 60 individuals with subacute SCI (<6 months postinjury). All participants will receive standard in-patient rehabilitation; the intervention group will additionally undertake a progressive ACET protocol for 8 weeks. Assessments will occur at baseline (T0), 4 weeks (T1), postintervention (T2) and 6-month follow-up (T3). Outcomes include static and dynamic sitting balance with kinematic measurements and high-density electromyography of the erector spinae, corticospinal excitability, muscle strength, functional independence and questionnaires of neuropathic pain, psychological well-being, self-efficacy and motivation, physical activity and health-related quality of life. Quantitative data will be analysed using mixed-model repeated measures ANOVAs and Student’s t-tests. Thematic analysis will be conducted on qualitative data obtained from focus groups in which the feasibility, enablers and challenges of ACET for individuals with subacute SCI will be discussed.

**Ethics and dissemination:**

This study was approved by The Health Research Authority and Health and Care Research Wales (22/NS/0054). Results will be published in peer-reviewed journals. Findings will be presented at National and International conferences for researchers and clinicians. Finally, results will be disseminated to the SCI community.

**Trial registration number:**

ISRCTN17247972

**STRENGTHS AND LIMITATIONS OF THIS STUDY**

- This multicentre, randomised controlled trial will evaluate trunk control over time in individuals with spinal cord injury (SCI) following a self-directed exercise programme, compared with those receiving standard care.
- The study assesses the broader impact of the intervention on individuals’ health and functioning, including impairments, activities, participation and environmental and personal factors.
- The study will use both quantitative and qualitative methods to enable multidimensional evaluation of patients’ responses to the intervention.
- A limitation is that neither the participants nor assessors are blinded to group allocation, which may introduce bias to the results.

## Introduction

 A spinal cord injury (SCI) damages synaptic connections between the corticospinal tract and motor neurons in the spinal cord, resulting in impaired volitional control of muscles and function below the site of injury.^1^ Globally, there are approximately 15.4 million people living with this devastating injury.^2^ In the United Kingdom alone, it is estimated that 104 000 people are living with SCI, with 4400 new cases reported every year.^3^

Trunk muscles are involved in a number of activities of daily living, such as reaching out for an object,^4^ moving the upper limbs^5^ and transfer.^6^ Most people with SCI have impaired trunk control which can compromise independence in daily activities, including stability, feeding and dressing.^7^,^10^ People in this condition may instead rely on their upper limbs to maintain stability or use compensatory postural control strategies,^4 11^ highlighting the importance of targeted trunk training to recover function and independence after SCI.

Research has suggested that upper-body exercise training improved trunk control in people with chronic SCI.^12^,^14^ For example, work showed improved dynamic sitting balance, accompanied by increased activity of the erector spinae (ES) and corticospinal excitability of the ES, after home-based, unsupervised ACET in individuals with chronic motor incomplete SCI.^12^ The underlying mechanisms could be related to use of shared neural circuits, such as crossed corticospinal facilitation, between the upper limbs and the trunk.^15 16^ This neural interaction has been shown to be preserved in people with incomplete SCI.^5^ Evidence has suggested that strengthening corticospinal connections in the spinal cord may lead to functional improvement in humans with chronic SCI.^17^,^19^

In the early stages following injury, research in animals with SCI showed sprouting of corticospinal axons rostral and caudal to the lesion site, forming alternative neural circuits to maintain supraspinal influence on motor neurons below the injury.^20^,^22^ Exercise interventions in rats with SCI increase the levels of neurotrophins that overcome myelin-derived axon growth inhibitors^23^ and promote synaptic plasticity in the spinal cord, creating new circuits to restore function.^24 25^ Similarly, evidence in humans with SCI has suggested the benefits of early motor training for promoting recovery of limb function.^26^,^28^ Given that neural pathways undergo extensive reorganisation during the early stages of SCI,^29^ particularly when the injury is incomplete,^30^ it is logical to hypothesise that the therapeutic effects of early ACET will be even greater in individuals with subacute SCI.

In addition to the physical benefits, exercise has been shown to improve pain and psychological symptoms in both general and clinical populations.^31 32^ People living with SCI experience higher levels of neuropathic pain,^33^ anxiety and depression compared with those without.^34^ However, low levels of exercise self-efficacy (ESE) in people with SCI^34^ on often decrease adherence and maintenance to exercise, thus hindering recovery.^35^

We are not aware of any studies investigating self-directed ACET and its effects on trunk control in individuals with subacute SCI. Hence, the primary aims of the study are to investigate the effects of ACET on volitional control of the trunk, and the neurophysiological mechanisms underpinning these effects in adults with subacute incomplete SCI. The secondary aims of the study are to evaluate short- and long-term benefits of the ACET on neuropathic pain, psychological well-being, and physical activity in this population. Additionally, self-efficacy and motivation of individuals with SCI for engaging in the ACET will be examined as potential predictors of adherence and the outcomes. This is important because people with SCI often experience higher levels of neuropathic pain,^33^ anxiety and depression compared with those without.^34^ However, low levels of ESE in people with SCI^34^ on often decrease adherence and maintenance to exercise, thus hindering recovery.^35^ Furthermore, we will investigate long-term translational effects of ACET on leisure time physical activity and health-related quality of life.

The objectives of the study are:

1. To assess static and dynamic sitting balance after 8 weeks of ACET in individuals with subacute incomplete SCI.
2. To examine training-induced changes in activation of the ES during static and dynamic sitting balance tasks and excitability of the corticospinal axons projecting to the ES in individuals with subacute incomplete SCI.
3. To evaluate neuropathic pain, psychological well-being, physical activity, independence, muscle strength and health related quality of life in individuals with a subacute incomplete SCI.
4. To predict adherence to the ACET and outcomes using individuals’ self-efficacy and motivation.

## Methods and analysis

### Study design

This study is a multicentre, two parallel-group, non-blinded, randomised controlled trial of a self-directed ACET in individuals with subacute SCI (<6 months postinjury). The study was approved by the North of Scotland Research Ethics Committee (1) (22/NS/0054) and prospectively registered on the International Standard Randomised Controlled Trial Number registry (ISRCTN17247972). This research is the second part of a larger trial which consists of a feasibility study (ISRCTN89333770; completed) and a randomised control trial (this study). The study will be conducted in accordance with the Declaration of Helsinki. The study protocol is reported following the SPIRIT checklist (online supplemental appendix 1).

### Patient and public involvement

Patient and public involvement group: Participants who took part in the feasibility study were involved in the protocol design. The new cohort will be involved in results interpretation and development of the next trial.

### Recruitment and consent process

Recruitment will be conducted at the Princess Royal Spinal Cord Injuries Centre (PRSCIC) and at the Yorkshire Regional Spinal Injuries Centre (YRSIC). Recruitment will be carried out by the medical team of the spinal units. Patients who meet the eligibility criteria will be approached by a member of the medical team and given a copy of the participant information sheet to read. They will be given at least 24 hours to consider their participation to the study. Prior to the consent process, potential participants will be given the opportunity to ask the research team questions regarding the study. The research team will obtain written informed consent from all participants prior to any data collection.

### Study population and sample size

The sample size was based on the effect size drawn from the feasibility study reporting an improvement in angular displacement of the trunk during forward reaching with ACET compared with the standard of care, with a large effect size (Cohen’s d = 0.85). An a priori power calculation suggested that a sample size of 46 patients with SCI, 23 in each group, is sufficient to detect a between-group difference for the primary outcome measure, using an independent t-test with 80% power and a 5% level of significance. To account for a 6-month follow-up after discharge, a 30% conservative dropout rate was considered. Hence, 60 participants in total will be recruited for this study.

### Eligibility criteria

The inclusion criteria are: (1) aged 18 years and above; (2) postinjury <6 months; (3) cervical or thoracic incomplete SCI; (4) able to sit without support for 30 s and (5) sufficient upper-limb function to voluntarily perform arm cycling movement on a stationary arm bike, with or without use of gripping aids.

The exclusion criteria are: (1) ongoing issues with shoulder instability or shoulder pain; (2) contraindications to exercise in an upright posture (eg, postural hypotension, unresolved pressure ulcer, uncontrolled cardiovascular conditions); (3) pregnancy and (4) unable to understand explanation of the study and/or instructions of the intervention.

### Interventions

All participants will receive standard in-patient rehabilitation care at the spinal unit. The ACET group will additionally undertake 8 weeks of progressive, unsupervised ACET with a stationary arm bike (MagneTrainer ER).

#### ACET intervention

A progressive protocol will be used in this study: 3 × 30 min interval training in weeks 1 and 2, 4 × 30 min interval training in weeks 3 and 4 and 5 × 30 min interval training in weeks 5 to 8. During all sessions, participants will be asked to maintain the rate of perceived exertion (RPE) between 4 and 6 out of 10 on the modified CR-10 Borg scale.^36 37^ This protocol was informed by our published study,^12^ and results of our feasibility study, which reported higher acceptance by individuals with subacute SCI to a progressive increase in the number of sessions per week. Additionally, it was suggested that patients may find interval arm cycling exercise more enjoyable compared with the continuous one. The first aim of the programme is to cycle at a cadence as close to 60 repetitions per minute (rpm) as possible and increase or decrease the resistance with the bike’s resistance control knob as needed to maintain the prescribed RPE, recognising it can take multiple sessions for participants to achieve this. Once at this level, participants are encouraged to choose other session plans to follow from a programme of seven interval sessions where cadence goes up to a 70–80 or 80–90 rpm range for short intervals before returning to 60 rpm (online supplemental appendix 2). The flexibility of session choice is aimed at promoting a sense of autonomy^38^ while still maintaining the prescribed intensity. Sessions are recorded by the participant (with help if needed by research team, hospital staff or friends and family) in their training diary, recording overall RPE for each session, with further detail of cadence adherence recorded from Garmin sensors (Garmin Edge, Cadence Sensor 2, Garmin, USA) placed on the crank of the arm bike. Thus, adherence will be analysed using both number of sessions performed across the 8 weeks (in relation to the programmed 34 sessions), and by similarity of actual cadence compared with programmed cadence of individual sessions. Prior to the ACET intervention, participants will engage in a 20-min initial conversation with the researchers (JK and MdRHM) aimed at enhancing adherence, using a systematic and integrated knowledge translation approach^39 40^ (see online supplemental appendix 3). Participants will undergo the first session of ACET with supervision from the researcher (JK or MdRHM) so that they can learn how to perform the exercise correctly and safely but will not be supervised for subsequent sessions, except some assistance in use of gripping aids from a ward nurse or physiotherapist. If a participant is discharged during the intervention, they will continue the intervention at home. For more information about the ACET intervention, see online supplemental appendix 4 TIDieR table.

### Assessments

The study has four assessment periods—baseline (T0), 4 weeks (T1), postintervention (T2), and 6 months follow-up (T3). Assessments from T0 to T2 are expected to occur while participants are still within 12 months postinjury. However, the T3 assessment may occur during the chronic phase of SCI for some participants. We expect that all participants will have the baseline and 4 weeks assessments conducted at the spinal units. We also anticipate some participants will have the postintervention and 6 months follow-up assessments at the spinal units or at the University of Birmingham, whichever is more convenient to them.

### Randomisation

**Figure 1:**
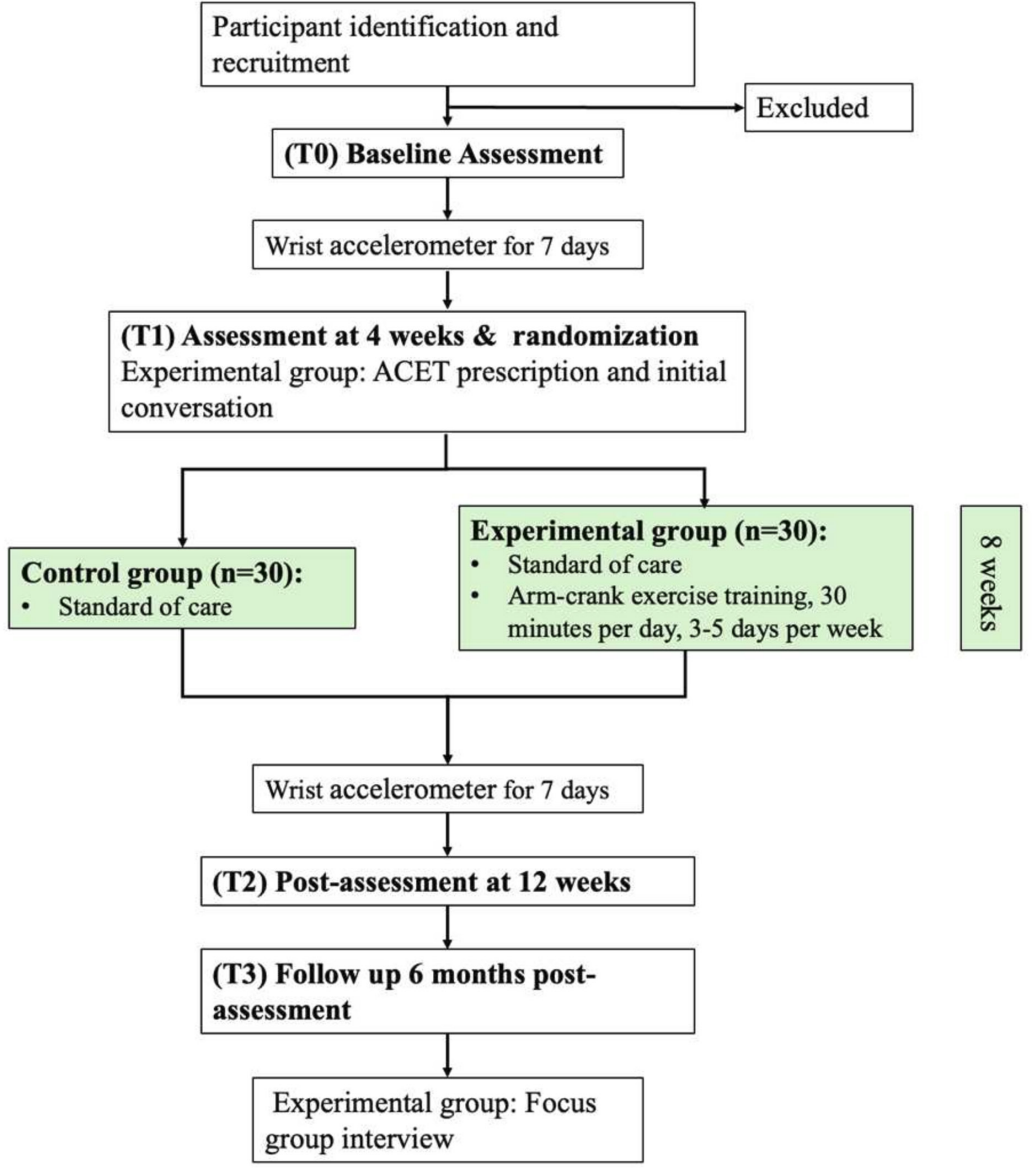
Trial flow chart. ACET: arm crank exercise training.

All participants will undergo one baseline assessment and one 4-week assessment to estimate individuals’ spontaneous recovery rate. After the 4-week assessment, participants will be randomly allocated (1:1) to an ACET group or a standard care control group using a minimisation randomisation^41^ to balance covariates of level of injury and classification of injury between groups. The procedure will start from the first participant being allocated to a study group at random. For each subsequent participant, we determine which study group would lead to better balance between the groups in the variables of interest. The randomisation will be performed by SYC who will not be involved in recruitment of participants. However, it is not achievable in this study to blind participants or researchers conducting the assessments given the type of the intervention and resources available. Figure 1 illustrates the trial flow chart.

### Outcomes

#### Demographics

Demographics (age, sex, height, weight, body mass index [BMI], ethnicity, postcode and level of education), and medical (eg, comorbidities) and treatment history of the participants will be recorded (cause of injury, duration, level of injury, severity of injury and sensory and motor scores according to the International Standards for Neurological Classification of Spinal Cord Injury (ISNCSCI)).^42^ Information regarding the duration and types of standard in-patient rehabilitation care will be collected from hospital records and patients’ exercise diaries during the trial. See table 1 for the assessment timeline. Detailed outcomes are listed below.

**Table 1:** Timeline for enrolment, interventions and assessments

		Study period					
		**Enrolment**	**Pre-allocation**	**Allocation**	**Postallocation**		
Timepoint		**−T0**	**T0**	**Allocation**	**T1**	**T2**	**T3**
Enrolment							
Eligibility screen		X					
Informed consent		X					
Allocation				X			
Intervention							
ACET						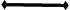	
Assessments							
Demographics, injury information	Questionnaire	X					
Independence	SCIM-III	X				X*	X
Sitting balance kinematics and HDEMG	Unsupported static sitting		X		X	X	X
	Forward reaching		X		X	X	X
	Lateral reaching		X		X	X	X
	Arm raise		X		X	X	X
	Rapid shoulder flexion		X		X	X	X
Corticospinal excitability	Motor evoked potentials		X		X	X	X
Motor recovery and impairment	ISNCSCI motor score and AIS	X	X		X	X*	X
Neuropathic pain	DN4		X		X	X	X
Depression	PHQ-9		X		X	X	X
Anxiety	GAD-7		X		X	X	X
Self-efficacy and motivation	TRSQ and PCS modified		X		X	X	X
Physical activity	GENEActive				X	X	
Leisure activity	LTPAQ-SCI						X
Quality of life	SF-36						X
ACET for early rehabilitation	Qualitative focus group						X

Note: (X*) SCIM-III and ISNCSCI at T2 or discharge.

ACET, arm-crank exercise training; AIS, American Spinal Injury Association Impairment Scale; DN4, Neuropathic pain four questions; GAD-7, general anxiety disorder; HDEMG, high-density surface electromyography; ISNCSCI, International Standards for Neurological Classification of Spinal Cord Injury; LTPAQ-SCI, leisure time physical activity questionnaire for people with spinal cord injury; PCS, perceived competence scales; PHQ-9, Patient Health Questionnaire; SCIM-III, The Spinal Cord Independence Measure; SF-36, 36-Item Short Form Survey; TRSQ, Treatment Self-Regulation Questionnaire.

#### Primary outcome measure

The primary outcome of the study is trunk control, assessed by:

*1. Volitional control of the trunk*

*Static and dynamic sitting balance*. Sitting balance will be assessed using the following tasks: unsupported sitting for 30 s, forward and lateral reaching,^12 43^ bilateral voluntary arm raise^44^ and rapid shoulder flexion.^12^ Participants will perform each task five times while seated on a plinth with their trunk unsupported, feet flat on the floor, hips and knees positioned at 90°, and the popliteal fossa approximately a palm’s width away from the edge of the plinth.

**Figure 2:**
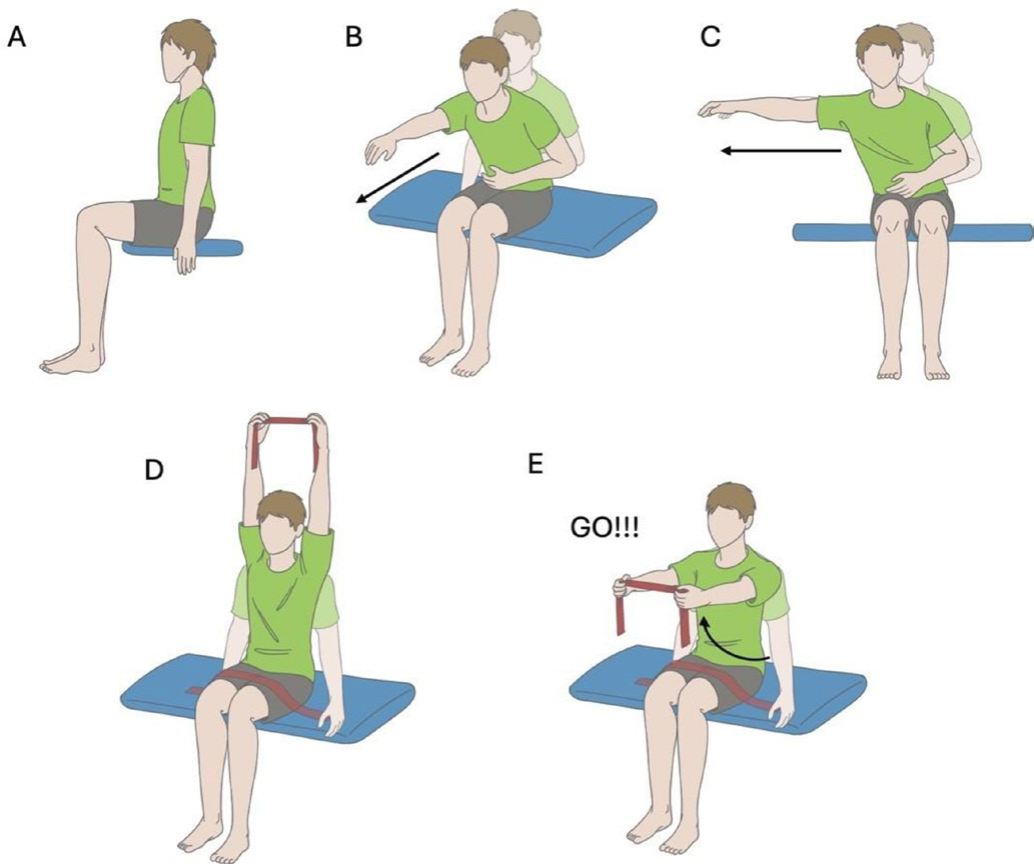
Assessment of volitional trunk control. (**A**) Unsupported sitting for 30 s; (**B**) forward reaching; (**C**) lateral reaching; (**D**) bilateral voluntary arm raise and (**E**) rapid shoulder flexion in response to a verbal ‘Go’ cue.

**Figure 3:**
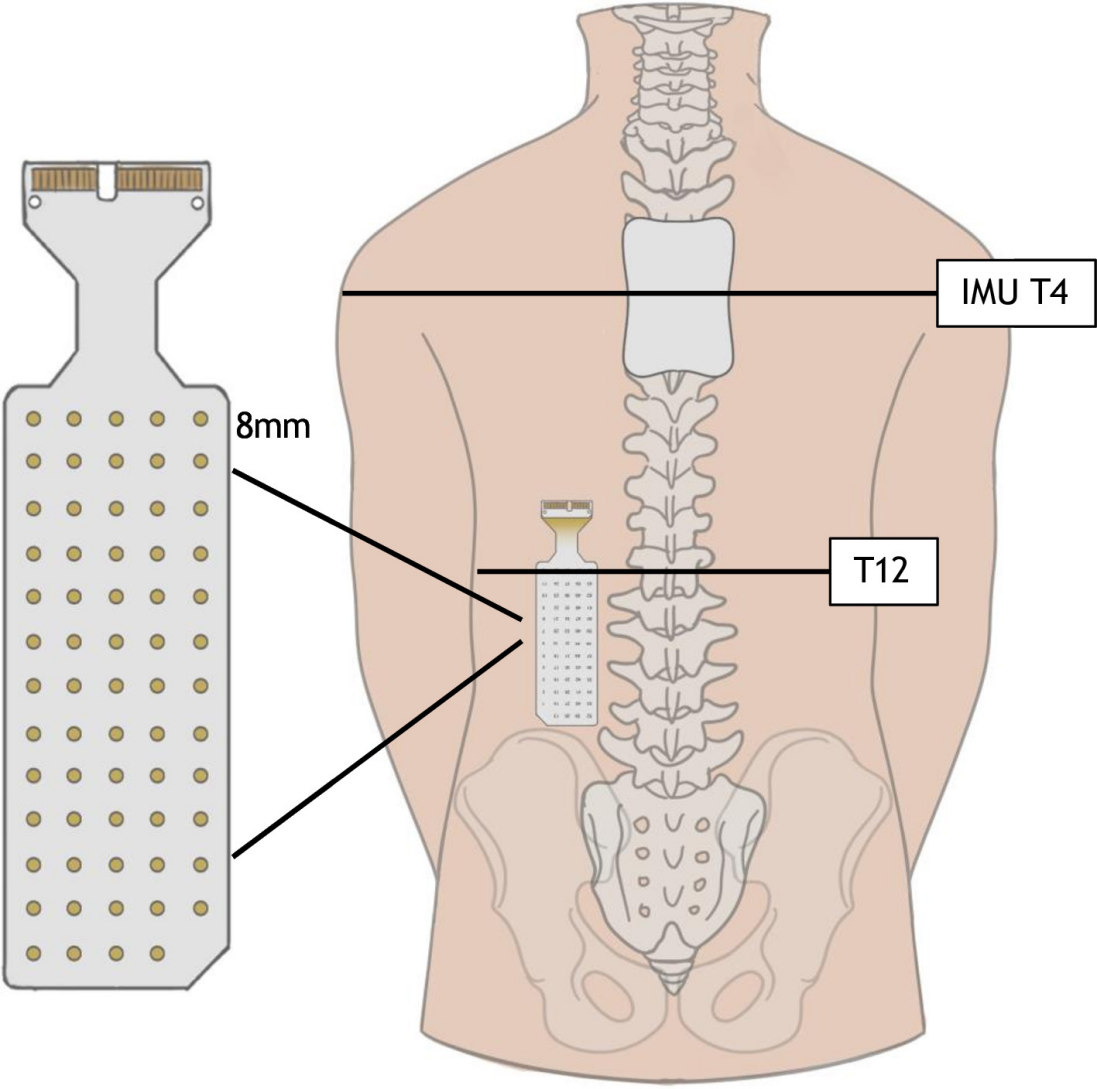
Setup of high-density surface electromyography. An inertial measurement unit (IMU) is placed at the 4th thoracic spinous process (**T4**) and an electrode grid is placed over the erector spinae parallel to the thoracic spine, with the top of the grid placed two centimetres laterally from the 12th thoracic spinous process (**T12**).

For the unsupported sitting task, participants will be instructed to sit unsupported as stable and upright as possible for 30 s (figure 2A). For the multidirectional reaching tasks (figure 2B and C), participants will be asked to sit in an upright position and use the less affected arm to reach forward and to the side as far as possible without losing balance. For the voluntary arm raise task (figure 2D), participants will be instructed to hold a resistance band with both hands and move the band up by flexing the shoulder joints to the maximum active range of motion. A rapid shoulder flexion task will be used to assess anticipatory and compensatory postural adjustments, whereby participants will, while sitting, raise their arms to a horizontal outstretched position as quickly as possible in response to a short, sharp verbal cue (figure 2E). Movement of the upper limbs and trunk will be captured in 2D videos recorded with a camera in 1080 p at 30 frames per second (iPad mini fifth generation, Apple, CA, USA). The camera will be placed at the angle perpendicular to the movement plane. Furthermore, the kinematics of the trunk will be recorded at 100 Hz using an inertial measurement unit (IMU)^45^; (Sessantaquattro+, OT Bioelettronica, Turin, Italy) placed at the fourth thoracic spinous process (figure 3). Sitting posture (spinal curvature at rest),^46^ reaching distance,^12^ angular displacement of the trunk^47^ and range of motion of the shoulder joints^44^ will be analysed from the 2D videos.

*2. Trunk control neurophysiological mechanisms*

*Neuromuscular control of the trunk*. Muscle activity of trunk muscles during seated balance tasks, as measured with high-density surface electromyography (HDEMG). Activity of the lumbar ES during seated balance tasks (figure 2) will be recorded using HDEMG which contains multiple small electrodes in a large electrode grid. HDEMG is a non-invasive technique and provides a topographic representation of muscle activity.^48 49^ This methodology is suitable for investigation of neuromuscular function of paraspinal muscles (ie, ES) which have multiple muscle fibres covering several levels of the spine and are innervated by multiple spinal nerves from the adjacent spinal levels.^50^ EMG recording from a larger area of the ES, as opposed to the bipolar EMG, may capture changes in regional distribution of activation across the spinal levels of the ES, providing new knowledge of recovery of trunk muscles in humans with SCI. Semi-disposable HDEMG grids (HD08MM1305, OT Bioelettronica) consisting of 64 oval-shaped electrodes arranged in 13 rows by 5 columns, minus the top left corner (figure 3; 1 × 1.5 mm electrode, 8 mm interelectrode distance), will be used to record lumbar ES activity. An adhesive foam (FO108MM1305, OT Bioelettronica) will be attached to the grid and its cavities will be filled with conductive paste (AC Cream, Spes Medica, Genoa, Italy). The skin over the muscle belly will be shaved, gently abraded with paste (Nuprep Skin Prep Gel, Weaver and Company, CO, USA), and cleaned with water to remove any residue from the paste. The grid will be placed on the ES in parallel with the lumbar spine, with the top edge of the grid placed 2 cm away from the centre of the 12th thoracic spinous process, and the bottom edge extended in the caudal direction to approximately the 4th lumbar spinous process (figure 3). One reference electrode (Ambu WhiteSensor WS, Ballerup, Denmark; 36 mm × 40 mm) will be placed on the seventh cervical spinous process and connected to the Sessantaquattro+, and a second ground electrode will be placed unilaterally over the iliac crest for a bipolar EMG system placed on the deltoid. An adhesive electrode (19.8 mm × 35 mm) of a bipolar EMG system (Delsys Bagnoli-2 EMG system) will be placed on the muscle bellies of the anterior deltoid (AD) of the less affected arm to record AD activity during the arm raised task. HDEMG will be recorded in monopolar mode with an amplifier (256 V/V gain; Sessantaquattro+, OT Bioelettronica) and digitised at 2000 Hz using a 16-bits A/D converter (10–500 Hz bandpass filter, input resistance 100 kΩ). Bipolar EMG (pre-amplified x1,000, band-passed filtered 20–450 Hz) will be recorded simultaneously via an isolated auxiliary input device (ISO-AUXSP, OT Bioelettronica), operating a 0.5 V/V gain alongside HDEMG using OT Biolab software (OT Bioelettronica) and stored in a local computer for postprocessing purposes. Root-mean-square (RMS) amplitudes and the centroid of RMS activation of the lumbar ES will be analysed.

*Corticospinal axons excitability of the ES*. To assess the excitability of the corticospinal axons projecting to the ES, high-voltage electrical stimulation (Digitimer stimulator, DS7AH, UK; square wave pulse, pulse duration 200 μs) will be applied at the cervicomedullary junction to elicit motor evoked potentials (CMEPs) in the ES^16 51^ when participants are seated in their preferred comfortable position. Between 10 and 15 CMEPs will be recorded and averaged. Participants will be asked to relax initially, but the Jendrassik manoeuvre will be introduced to assess if little or no response in the ES is observed. The Jendrassik manoeuvre, where a participant will clench their teeth and flex fingers in a hook shape, has been shown to facilitate corticospinal excitability.^52^ Peak-to-peak amplitudes and latency of the averaged CMEP will be calculated.

#### Secondary outcomes

*3. Injury classification*

The American Spinal Injury Association Impairment Scale (AIS)^42^ grades (A to E) will be documented to determine any improvement in sensory/motor preservation after the intervention, relative to standard of care alone.

*4. Motor recovery*

Strength will be assessed by a physiotherapist using the ISNCSCI^42^ upper extremities motor score and lower extremities motor score.

*5. Neuropathic pain*

Neuropathic pain will be documented using the Neuropathic Pain 4 Questions (DN4) questionnaire that rates from 0 to 10, being ≥4 suggestive of neuropathic pain.^53^

*6. Physical and mental health*

Depression and anxiety will be assessed using the Patient Health Questionnaire (PHQ-9)^54^ and Generalised Anxiety Disorder (GAD-7),^55^ respectively. Each questionnaire rates from 0 to 21, with a classification of mild (0–5 points); moderate (6–10 points); moderately severe (11–15 points) and severe (15–21 points).

*7. Self-efficacy for engagement in exercise*

Participants will complete a modified version of the perceived competence scales (PCS),^56^ a short assessment of ACET self-efficacy, with responses indicated on a scale from 0 to 10, with 0 = no confidence and 10 = completely confident.

*8. Motivation for exercise engagement*

A modified version of the Treatment Self-Regulation Questionnaire (TRSQ)^57^—comprising 16 questions on a scale from 1 (not at all true) to 5 (very true)—will gauge a participant’s source of motivation for why they might want to engage in ACET (eg, to impress family or friends or to improve health). See online supplemental appendix 5 for the self-efficacy and motivation scales.

*9. Physical activity levels*

Free-living physical activity energy expenditure will be calculated from raw acceleration signals recorded using a wrist-worn accelerometer (GENEActiv) for prior to T1 and after T2. The device has been validated in wheelchair users,^58^ and participants will be instructed simply to wear the device continuously for 7 days. Additionally, to understand whether the early ACET may lead to a more active lifestyle in the long term, the Leisure Time Physical Activity in People with SCI questionnaire (LTPAQ-SCI) will be used at the follow-up to record the time spent performing moderate-to-vigorous-intensity leisure time physical activities.^59^ Research has shown that the number of minutes spent per week performing moderate-to-vigorous intensity activity is positively correlated with cardiorespiratory fitness.^60^ Pre-injury physical activity levels will be documented during the pre-intervention conversation. However, a more accurate measure of physical activity prior to the injury is not feasible to be collected as part of this study.

*10. Health-related quality of life*

The Short Form (SF-36) walk-wheel Health Questionnaire (V.2)^61^ includes eight scales, which together produce two main summary measures, each ranging from 0 to 100: physical health and mental health. The physical health measure includes scales of physical functioning, role-physical, bodily pain and general health. The mental health measure includes vitality, social functioning, role-emotional and mental health.^62^

*11. Independence*

The Spinal Cord Independence Measure (SCIM-III) scale^63 64^ will be assessed at admissions and discharge to indicate functional independence of the participants.

#### Qualitative methods

*12. Focus group*

We will conduct online semi-structured focus groups with a purposive sample of 15 participants from the ACET group to discuss the content and delivery of the intervention. Each group will have up to five participants and be moderated by an experienced qualitative researcher (MdRHM). We will explore participants’ expectations and experiences of the intervention, barriers and enablers to participation, and views on future implementation (online supplemental appendix 6). Discussions will be audio recorded and transcribed verbatim. The focus group recordings will be coded using Nvivo 9 software, analysed using a deductive thematic analysis^65 66^ and will be interpreted together with the quantitative data.

### Statistical analyses

Statistical analyses will be performed using the latest version of SPSS available at the time of analysis (IBM, Armonk, NY, USA). The distribution of the continuous variables will be tested for normality using the Shapiro-Wilk test and appropriate descriptive parameters will be reported and statistical tests will be applied accordingly. A mixed-model two-way repeated measures ANOVA, with group as the between-participant factor and time as the within-participant factor, will be used to compare outcome measures. This analysis will be performed according to the intention to treat approach. Post hoc tests will be applied where there is a main effect. Significance level will be set at 0.05 for all statistical tests. For the data that are normally distributed, effect sizes of partial eta squared (η2p) or Cohen’s d will be calculated. Additionally, potential confounders (eg, age, days since injury) will be included in the analysis. Subgroup analyses (eg, based on AIS classification) will also be performed if appropriate.

### Safety

Exercise intervention and neurophysiological and behavioural assessments are considered as of low risk. Potential risks relating to the study participation are stated in the participant information sheet and will also be verbally explained to the potential participant to ensure that they are fully informed before providing written consent. Any study-related adverse or serious adverse events will be documented and reported in accordance with the Sponsor’s safety reporting procedures.

### Data management

Information with regards to study participants will be kept confidential and managed in accordance with the Data Protection Act 2018, General Data Protection Regulation (GDPR), The UK Policy for Health and Social Care and Research Ethics Committee Approval. Data generated by this study will be kept for 10 years as per University of Birmingham guidelines including the patient consent forms. Data will be stored on secure, encrypted university servers and accessed only by the research team using 2-factor authorisation. No data will be stored on laptops or portable drives.

## Discussion

Evidence generated from this study may influence the current in-patient rehabilitation care post-SCI in the National Health Service in the United Kingdom. Trunk rehabilitation is important for regaining function and independence of individuals after SCI, but it requires high levels of input and supervision from professionals, reducing the time that patients can spend on the rehabilitation, thereby hindering recovery trajectory. Our ACET programme is a self-directed intervention and uses simple equipment that can be easily set up in a clinical environment. Our programme, therefore, has the advantage of being simply delivered alongside the standard of care. Using the comprehensive outcome measurements our study will address the effects of the intervention, but also the mechanisms underpinning the effects of the intervention, and long-term benefits to the individuals with SCI. Moreover, this study will provide insight into factors promoting participation in such an exercise intervention as well as barriers to engagement that are important for optimising adherence to future exercise interventions in individuals with SCI. Taken together, the results have the potential to contribute to the development of personalised treatment plans for recovery after SCI and to provide broader implications for trunk rehabilitation in other clinical populations, such as stroke survivors, amputees and ageing.

## Supplementary material

10.1136/bmjopen-2024-092226 online supplemental file 1

10.1136/bmjopen-2024-092226 online supplemental file 2

10.1136/bmjopen-2024-092226 online supplemental file 3

10.1136/bmjopen-2024-092226 online supplemental file 4

10.1136/bmjopen-2024-092226 online supplemental file 5

10.1136/bmjopen-2024-092226 online supplemental file 6
