# Supplementary material for: Self-directed arm-crank exercise to improve volitional control of the trunk in patients with subacute spinal cord injury: a multicentre, parallel-group, randomised controlled trial protocol
Source: BMJ Open. 2025 Aug 21;15(8):e092226. doi: 10.1136/bmjopen-2024-092226 (PMC12374656; doi:10.1136/bmjopen-2024-092226)

RPM Study: Arm-cycling sessions

Start by working up to 30 minutes continuous cycling (session 1). Then pick any session on this sheet and tick off every time you complete it.

Complete each session no more than 5 times to ensure variety, and save the later sessions till further on in the 8 weeks as sessions get more difficult moving from 1 to 7. Record RPE in your training diary.

Remember: 3 sessions per week weeks 1 & 2, 4 per week for weeks 3 & 4, then 5 weekly.

Session 1

Completed: ○ ○ ○ ○ ○

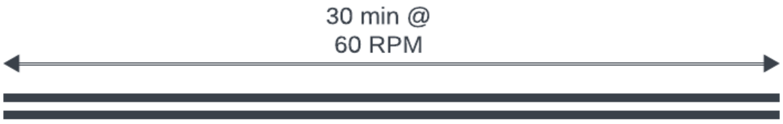

Session 2

Completed: ○ ○ ○ ○ ○

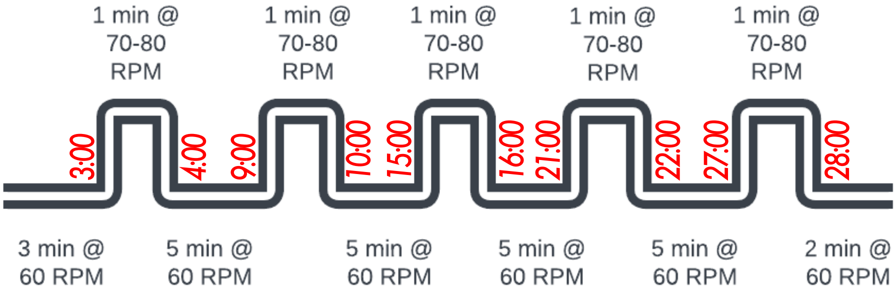

Session 3

7:00 – up
8:30 – up
9:30 – down
11:00 – down
18:00 – up
19:30 – up
20:30 – down
22:00 – down

Completed: ○ ○ ○ ○ ○

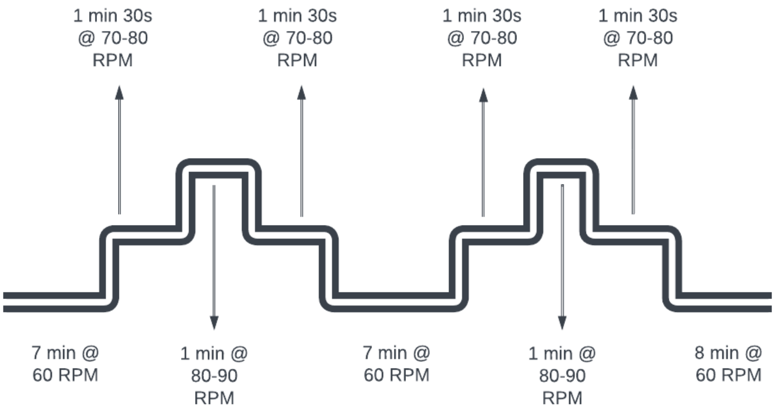

Session 4

Completed:

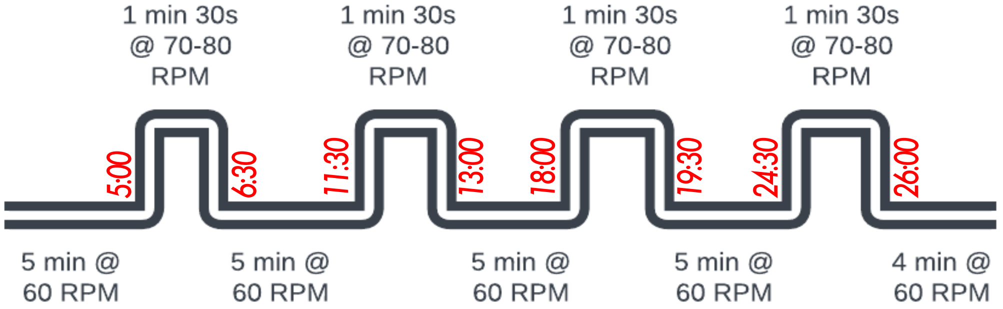

Session 5

Completed:

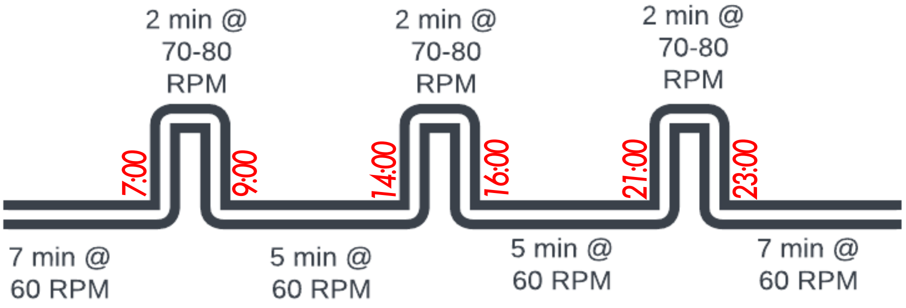

Session 6

Completed:

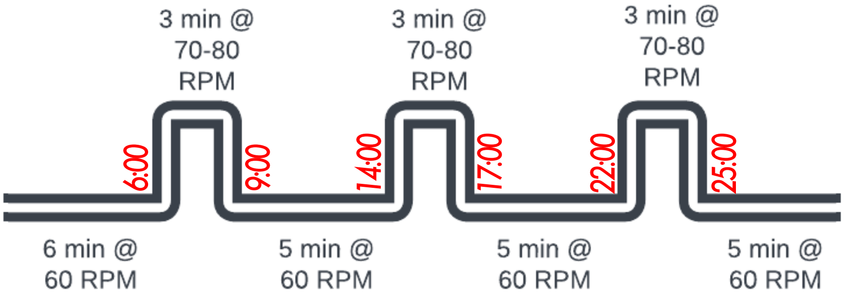

Session 7

Completed:

4:30 – up
6:00 – up
7:00 – down
8:30 – down
13:00 – up
14:30 – up
15:30 – down
17:00 – down
21:30 – up
23:00 – up
24:00 – down
25:30 – down

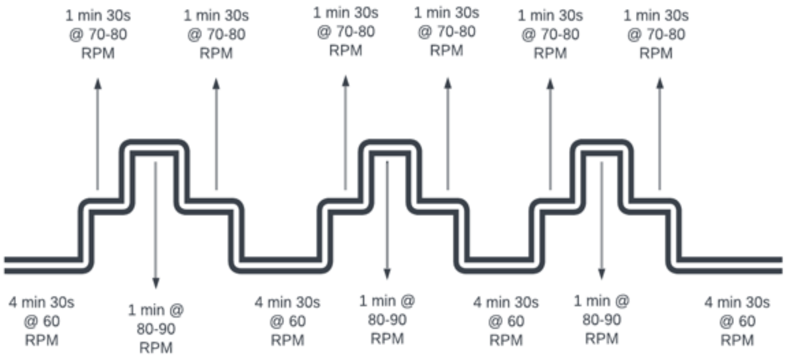

Supplement: online supplemental file 2 [file bmjopen-15-8-s002.pdf]
