# Supplementary material for: Self-directed arm-crank exercise to improve volitional control of the trunk in patients with subacute spinal cord injury: a multicentre, parallel-group, randomised controlled trial protocol
Source: BMJ Open. 2025 Aug 21;15(8):e092226. doi: 10.1136/bmjopen-2024-092226 (PMC12374656; doi:10.1136/bmjopen-2024-092226)
Supplement: online supplemental file 3 [file bmjopen-15-8-s003.pdf]

ID:
Date:

SCI Initial conversation

Self-efficacy and motivation questionnaire follow-up for those in the intervention group

TIME POINT: WEEK 4 ASSESSMENT, JUST BEFORE INTERVENTION

Ask permission: **Based on your responses to the self-efficacy and motivation questionnaire, I will ask some follow up questions and, if you'd like, we can create an action plan together. Is that alright?**

Question 1: **What has your experience been with exercise and physical activity up until now?**

Question 2: **What has your experience been with rehabilitation or forms of therapeutic exercise?**

ID:
Date:

Statement 1 and follow up: **You rated your confidence in your ability to do the arm-cycling programme at ___ and your confidence that you will regulate yourself to perform it at ___.**
What are some of the barriers relating to your capabilities, environment and opportunities?

Goal setting: **Let's set some short term (2 month) and long term (6 month) goals.**

Then: **How are you going to achieve these? What do you have in mind? Can I offer any tips? (What do you need from me?) Can I check in with you next week, at discharge and at programme end?**

Finally, **when and where will you do your first session(s)?**
