# Supplementary material for: Self-directed arm-crank exercise to improve volitional control of the trunk in patients with subacute spinal cord injury: a multicentre, parallel-group, randomised controlled trial protocol
Source: BMJ Open. 2025 Aug 21;15(8):e092226. doi: 10.1136/bmjopen-2024-092226 (PMC12374656; doi:10.1136/bmjopen-2024-092226)
Supplement: online supplemental file 4 [file bmjopen-15-8-s004.docx]

Appendix 4. TIDieR Intervention Group.

| **TIDieR item** | **Intervention group** |
| --- | --- |
| **1.Provide the name or a phrase that describes the intervention.** | Arm-crank ergometry training (ACET) for the trunk function for people living with an incomplete spinal cord injury (SCI). |
| **2.Describe any rationale, theory, or goal of the elements essential to the intervention.** | To increase the intensity of trunk rehabilitation in people with a subacute SCI in the hospital setting, we need to find ways of providing interventions that are effective, safe and does not require supervision/minimal supervision from the therapist. ACET is a simple and self-directed rhythmic exercise where the arms are used to spin. ACET is widely used to improve the cardiorespiratory system in people with SCI, and recently studies have found potential benefits for improving trunk control in people with SCI^1^. |
| **3.Materials: Describe any physical or informational materials used in the intervention, including those provided to participants or used in intervention delivery or in training of intervention providers. Provide information on where the materials can be accessed (e.g. online appendix, URL).** | - Arm bike: The arm bike (MagneTrainer ER) can be placed on a standardised hospital bedside table, allowing the participants to perform the exercise in the ward. Wheel stoppers will be used to prevent the table moving during the exercise. If the participant does not have sufficient grip function, the participant will be given a bandage or active grip to hold pedals. - Initial conversation. - Exercise calendar diary to annotate sessions per week, minutes per session, and fatigue levels using the modified CR-10 Borg scale. |
| **4.Procedures: Describe each of the procedures, activities, and/or processes used in the intervention, including any enabling or support activities.** | - Initial conversation (20-minutes): As a strategy to improve adherence and self-efficacy, all participants in the ACET group will have an initial conversation prior to the first ACET session. A worksheet consisting of 5 open questions for facilitation of conversation have been developed to tailor to the needs of the study. Additionally, the research team will contact the participants after a week into the intervention to check if they need further support for carrying out the planned exercise. - ACET familiarization and prescription (20-minutes): Participants will be instructed to do the arm cycling while seated on a flat and steady surface with the trunk up right and the elbows almost fully extended when the pedal is further away from the participant´s body. The position of the participant matching the bike pedal axis with the participant height of the shoulders. - During the ACET programme: Participants in the ACET group will undertake an arm-crank exercise training programme for 8 weeks. If needed, during the program, hospital staff members and families/carers will help with the preparation of the session and end of the session (e.g, place the hand in the pedal with the active hand, adjust the position of the participant matching the bike pedal axis with the participant height of the shoulders). - Members of the hospital staff might also need to help with the recording of exercises diaries if participants unable to write. |
| **5.For each category of intervention provider (e.g. psychologist, nursing assistant), describe their expertise, background and any specific training given.** | - Initial conversation: The researchers who will conduct the initial conversation (JK, MHM) completed the SCI Physical Activity Counselling Training from the University of British Columbia  (https://sciactioncanada.ok.ubc.ca/sci-physical-activity-counselling/) which was designed based on a systematic and integrated knowledge translation approach^2,3^, aimed at promoting physical activity in adults with SCI. - ACET familiarization and prescription: All researchers who will participate in the study are experts in exercise and/or rehabilitation. - During the 8 weeks program: all hospital staff members that will help in the study are experts in SCI rehabilitation. If participants are discharged home before the end of the intervention, all families/carers that will help in the study will be shown how to help the participant. |
| **6.Describe the modes of delivery (e.g. face-to-face or by some other mechanism, such as internet or telephone) of the intervention and whether it was provided individually or in a group.** | - Face-to-face delivery (provided individually): Initial conversation, ACET familiarization and prescription session. - Self-administered: 8-week ACET programme. |
| **7.Describe the type(s) of location(s) where the intervention occurred, including any necessary infrastructure or relevant features.** | The intervention will occur in a hospital setting (SCI units). If participants are discharged from hospital before the intervention is finished, participants will continue the intervention in their own homes. |
| **8.Describe the number of times the intervention was delivered and over what period of time including the number of sessions, their schedule, and their duration, intensity or dose.** | 8 weeks Arm-crank exercise training programme: 3 x 30 minutes per week for week 1-2; 4 x 30 minutes/week for week 3-4; 5 x 30 minutes/week for week 5-8 in an upright seated posture. Exercise intensity will be maintained at a perceived exertion of 4-6 out of 10 using the modified CR-10 Borg scale.  Participants are encouraged to do 30 minutes per session accounting for their perceived exertion and/or pain. |
| **9.If the intervention was planned to be personalised, titrated or adapted, then describe what, why, when, and how.** | - Participants who can sit without back support will be instructed to perform ACET in an upright seated posture to activate the trunk muscles throughout. Individuals with more significant impairments in trunk control would be encouraged to gradually increase the duration without back support in each session to enhance activation of the trunk muscles. - As the perceived exertion is different for each person, participants will be asked to maintain a perceived exertion of 4-6 out of 10 (modified Borg scale). - If participants are unable to use both arms, they will do the ACET with one arm. - If a participant is discharged during the 8-weeks intervention, they will be instructed to take the arm bike home and continue the exercise at home. Additional support will be available to them, such as instructions on how to set up the arm bike at home via video calls. Furthermore, if a participant develops a new medical condition, such as a urinary infection or pressure ulcers, necessitating suspension of the intervention, a 'catch-up' period will be provided to allow the participant to regain their physical level as to before the onset of the condition based on the RPE and exercise duration. After regaining the physical capacity, the participant will continue the remaining exercise sessions. However, if the interruption of the intervention is longer than 4 weeks (>50% of the planned intervention period), the participant will be withdrawn from the study as their body function may have changed in the past four weeks. - Participants can perform the exercise program at any time at their own convenience. Once participants are able to do 30 minutes of ACET with no resistance at 60 revolution per minute (rpm), changes in speed and resistance are freely chosen by the participants so they are more “in charge” of their treatment. |
| **10.If the intervention was modified during the course of the study, describe the changes (what, why, when, and how).** | To this date, there have been no modifications. |
| **11.Planned: If intervention adherence or fidelity was assessed, describe how and by whom, and if any strategies were used to maintain or improve fidelity, describe them.** | The initial conversation is a strategy for adherence, and it’s been done by the researchers.  Participants will record the number of ACET sessions and the minutes per session in a weekly calendar. |
| **12.Actual: If intervention adherence or fidelity was assessed, describe the extent to which the intervention was delivered as planned.** | To be explored at the end of the study. |

Appendix 4 References

1 van Helden, J. F. L. *et al.* Home-based arm cycling exercise improves trunk control in persons with incomplete spinal cord injury: an observational study. *Sci Rep* **13**, 22120 (2023). <https://doi.org:10.1038/s41598-023-49053-w>

2 Hoekstra, F. *et al.* Theory- and evidence-based best practices for physical activity counseling for adults with spinal cord injury. *J Spinal Cord Med*, 1-13 (2023). <https://doi.org:10.1080/10790268.2023.2169062>

3 Milne, S., Orbell, S. & Sheeran, P. Combining motivational and volitional interventions to promote exercise participation: protection motivation theory and implementation intentions. *Br J Health Psychol* **7**, 163-184 (2002). <https://doi.org:10.1348/135910702169420>
