# Supplementary material for: Self-directed arm-crank exercise to improve volitional control of the trunk in patients with subacute spinal cord injury: a multicentre, parallel-group, randomised controlled trial protocol
Source: BMJ Open. 2025 Aug 21;15(8):e092226. doi: 10.1136/bmjopen-2024-092226 (PMC12374656; doi:10.1136/bmjopen-2024-092226)
Supplement: online supplemental file 5 [file bmjopen-15-8-s005.pdf]

Motivation and Self-Efficacy Questionnaire

Study ID:

Date:

Assessment time point: T0/T1/T2/T3

SELF EFFICACY (TASK and SELF REGULATION)

For each question below, please indicate (by circling the number) your level of confidence at the present time on a scale from 1 (no confidence) to 10 (complete confidence).

1. *How confident are you* that you are able to do the arm crank exercise at a moderate intensity?

   0 1 2 3 4 5 6 7 8 9 10
   not at all confident … totally confident

2. *How confident are you* that you will regulate yourself to do the arm crank exercise for 3-5 days per week (30 minutes each time)?

   0 1 2 3 4 5 6 7 8 9 10
   not at all confident … totally confident

MOTIVATION TO ENGAGE IN THE ARM-CRANK EXERCISE

On the 5 point scale ranging from 1 (not at all true) to 5 (very true), please circle the number which best describes how you feel about the arm-crank exercise.

I intend to engage in the arm-crank exercise programme:

1. Because I feel that I want to take responsibility for my own health.

   1 (not at all true) **2** **3** (somewhat true) **4** **5** (very true)

2. Because I would feel guilty or ashamed of myself if I did not.

   1 (not at all true) **2** **3** (somewhat true) **4** **5** (very true)

3. Because I personally believe it is the best thing for my health and functioning.

   1 (not at all true) **2** **3** (somewhat true) **4** **5** (very true)

4. Because my *medical team* would be upset with me if I did not.

1	2	3	4	5
not at all true		somewhat true		very true

5. Because *my family/close fri*ends would be upset with me if I did not.

1	2	3	4	5
not at all true		somewhat true		very true

6. Because I have carefully thought about it and believe it is very important for many aspects of my life going forward.

1	2	3	4	5
not at all true		somewhat true		very true

7. Because I would feel bad about myself if I did not.

1	2	3	4	5
not at all true		somewhat true		very true

8. Because it is an important choice I really want to make.

1	2	3	4	5
not at all true		somewhat true		very true

9. Because I feel pressure from *my medical team* to do so.

1	2	3	4	5
not at all true		somewhat true		very true

10. Because I feel pressure from *my family/close fri*ends to do so.

1	2	3	4	5
not at all true		somewhat true		very true

11. Because it is consistent with my life goals now.

1	2	3	4	5
not at all true		somewhat true		very true

12. Because I want *my medical team* to approve of me.

1	2	3	4	5
not at all true		somewhat true		very true

13. Because I want *my family/close friends* to approve of me.

1	2	3	4	5
not at all true		somewhat true		very true

14. Because it is very important for being as healthy and functional as possible.

1	2	3	4	5
not at all true		somewhat true		very true

15. Because I want *my medical team* to see I can do it.

1	2	3	4	5
not at all true		somewhat true		very true

16. Because I want *my family/close friends* to see I can do it.

1	2	3	4	5
not at all true		somewhat true		very true
