# Supplementary material for: Self-directed arm-crank exercise to improve volitional control of the trunk in patients with subacute spinal cord injury: a multicentre, parallel-group, randomised controlled trial protocol
Source: BMJ Open. 2025 Aug 21;15(8):e092226. doi: 10.1136/bmjopen-2024-092226 (PMC12374656; doi:10.1136/bmjopen-2024-092226)
Supplement: online supplemental file 6 [file bmjopen-15-8-s006.docx]

**Early self-initiated upper-body exercise to improve volitional control below the level of injury after spinal cord injury**

Focus group schedule.

RECORDING

1. **Introduction by Moderator (5 minutes)**

Thank you all for joining us today. We really appreciate your time and willingness to share your experiences from the "Arm cycling study”. Your feedback is invaluable in helping us understand what worked well and how we can improve the programme. The purpose of this session is to hear your thoughts on participating in the 8-weeks training programme. There are no right or wrong answers—we’re here to learn from your experiences. The discussion will take about 60 minutes and will be recorded to ensure we capture everything accurately.

**To protect your privacy:**

- Your names will not appear in any reports.
- If we use quotes, we’ll use pseudonyms.
- The recordings will only be accessible to the research team, stored securely on the University research data server for 10 years, and then deleted.

**A few ground rules before we begin:**

- Please avoid using real names during our discussion; if you do, we’ll replace them with pseudonyms in the transcript.
- Let’s aim for a respectful and collaborative conversation. It’s okay to agree or disagree, but please allow one person to speak at a time so everyone can share their thoughts.

Now, let’s start by introducing ourselves. Please share your first name or a nickname you’re comfortable using today.

Before we continue, I’d like to confirm that you all agree to keep this discussion confidential and that you’re happy to proceed. If so, please raise your hand to indicate your consent.

Thank you! I’ll now begin the recording.

1. **Arm-Crank Exercise Training Experience**
2. **Trunk control**

- Do you think core/trunk control is important for you?
- How relevant is trunk control for your recovery?
- Since your injury, what exercises have you been doing to improve trunk control?

1. **Expectations before the study**

- What did you expect when you first agreed to participate in this study?
- Do you feel that the study met these expectations?

– If yes, in what way did it meet your expectations?
– If no, what was different to what you had expected?

1. **Overall Experience**
   - What was your overall experience with the arm cycling exercise programme?
   - What aspects of the programme did you enjoy the most?
   - Were there any parts of the programme that you found challenging or didn’t enjoy?
2. **Enables and Barriers to Participation**
   - How easy or difficult was it to follow the **exercise protocol?** Were there any aspects that facilitated your ability to do the exercise? Are there any aspects that could be improved?"
   - What challenges did you face while doing the arm cycling programme, both in the hospital and at home? (*Prompt: In the hospital, was it space limitations, fatigue, or something else? At home, was it difficult to set up the arm bike, fitting exercise into your routine or feeling overwhelmed?)*
3. **Self-Directed Exercise During Recovery**
   - Since the programme was unsupervised, how did you feel about doing additional exercises on your own in the early stages of your injury?
   - Did you feel confident in knowing what to do, or were there times when you needed more guidance?
   - If you ever felt unsure about the exercises, do you think it affected how often or how well you participated in the programme?
4. **Motivation & Adherence Challenges**
   - Did you have any motivators to do the arm cycling exercise?
   - Some participants initially felt confident about the programme but found it challenging to maintain consistency over time. Did you have the same experience?
   - What factors do you think influenced this, and what might help improve adherence (*e.g., family encouragement, ease of use, enjoyable aspects like music, pre-injury fitness level)*?
   - Are there any strategies that could make the programme more engaging or easier to follow?
5. **Mental and Physical Impact**
   - Did the exercise have any effect on your physical recovery? If so, in what way?
   - Did the exercise have any impact on your mental health or emotional well-being?
   - Would you recommend this type of exercise to other patients in the hospital? Why or why not?
6. **Future Implementation**
7. **Continuing Arm-Crank Exercise After Hospital Discharge**
   - How do you feel about incorporating arm cycling into your routine as a regular form of exercise or sport? What factors would make you more or less likely to continue doing it over time?
   - If you were to continue this exercise regularly, how many sessions per week do you think would be ideal for you?
   - In what settings do you think this type of exercise would be most accessible and beneficial after discharge? (*Prompt: At home, in community gyms, in a private physio*)?
8. **Incorporating Arm Cycling into Standard Rehabilitation**
   - What are your thoughts on including unsupervised arm cycling in hospital rehabilitation? In what situations do you think it would or wouldn’t be beneficial?
   - Based on your experience, at what stage of recovery do you think this type of exercise would be most useful, if at all? *(Prompt: Early recovery (acute phase), during rehabilitation (subacute phase), or later in long-term recovery (chronic phase)*?
9. **Closing (5 minutes)**

Before we wrap up, I’d like to ask:

- Is there anything we haven’t discussed that you would like to add and that is important for us to know about the experiences of participating in this programme?
- If you could change one thing about the programme, what would it be?

Thank everyone for their time and useful participation.
