# Supplementary material for: Self-directed arm-crank exercise to improve volitional control of the trunk in patients with subacute spinal cord injury: a multicentre, parallel-group, randomised controlled trial protocol
Source: BMJ Open. 2025 Aug 21;15(8):e092226. doi: 10.1136/bmjopen-2024-092226 (PMC12374656; doi:10.1136/bmjopen-2024-092226)
Supplement: Supplementary file 7 [file bmjopen-2024-092226.reviewer_comments.pdf]

PEER REVIEW HISTORY

BMJ Open publishes all reviews undertaken for accepted manuscripts. Reviewers are asked to complete a checklist review form and are provided with free text boxes to elaborate on their assessment. These free text comments are reproduced below.

ARTICLE DETAILS

Title (Provisional)

Self-directed arm-crank exercise to improve volitional control of the trunk in patients with subacute spinal cord injury: a multicentre, parallel-group, randomised controlled trial protocol.

Authors

Hidalgo Mas, Maria del Rocio; Kearney, Joshua; Middleton, Vicki; Chiu, Chuang-Yuan; Duda, Joan; Nightingale, Thomas; Martinez-Valdes, Eduardo; Ahmed, Zubair; Chiou, Shin-Yi

---

VERSION 1 - REVIEW

**Reviewer**	**1**
**Name**	**Quinzaños-Fresnedo, Jimena**
**Affiliation**	**Instituto Nacional de Rehabilitación, Neurologic Rehabilitation**
**Date**	**06-Dec-2024**
**COI**	**None**

Thank you for the opportunity to read and review this interesting and original article.

Its main strength is its potential impact on spinal cord injury health. Additionally, the methodological approach is appropriate, which ensures the project's internal and external validity.

However, some concerns must be assessed to improve the project.

Strengths and limitations of the study

Since you are presenting a protocol with no results, you cannot affirm that the study “reveals” nor “provides”, please consider changing this to “could reveal” or “has the potential to provide”.

Introduction

Consider changing the order of the paragraphs. You start with clinical data, then discuss pathophysiological mechanisms, afterward you return to clinical data, and then again to

physiological data. Finally, you present clinical data unrelated to trunk control. The information provided is important to demonstrate the significance of the work, but it could be better organized to facilitate reading.

Objective 3: Please consider other clinical, validated data to evaluate improvements (strength, independence, trunk control). Some of them are mentioned later in the manuscript (strength).

Eligibility criteria: did you consider that subjects with more than 4 months since the injury will complete the intervention in a chronic phase?

Please justify why you will only include incomplete injuries.

Exclusion criteria: Did you consider cardiovascular health (previous co-morbidities).

Interventions

Please specify the time lapse between the discharge and initiation of the intervention.

Outcomes

Demographics. Please consider adding previous physical activity and comorbidities.

The SCIM-III (and not SCIM-3) is not a questionnaire but a disability scale. A self-reported version exists, the SCIM-SR (Fekete C, Eriks-Hoogland I, Baumberger M, Catz A, Itzkovich M, Lüthi H, Post MWM, von Elm E, Wyss A, Brinkhof MWG. Development and validation of a self-report version of the Spinal Cord Independence Measure (SCIM III). Spinal Cord. 2013 Jan;51(1):40–7).

Volitional control of the trunk. Please consider the following paper for the IMUs analysis: Pérez-Sanpablo, A. I., Quinzaños-Fresnedo, J., Romero-Ixtla, M., Aguirre-Güemez, A. V., Rodríguez-Reyes, G., Pérez-Zavala, R., … Quijano-González, Y. (2021). Validation of inertial measurement units for the assessment of trunk control in subjects with spinal cord injury. The Journal of Spinal Cord Medicine, 46(1), 154–163. https://doi.org/10.1080/10790268.2021.1975083.

Secondary outcomes.

3. The international standards for neurologic classification of spinal cord injury (ISNCSCI) have multiple outcome measures. The motor function is divided into upper extremities motor score (UEMS), and lower extremities motor score (LEMS). Please use the right terms and specify if you will use both or only one.

Since trunk control also depends on afferents (and those pathways could benefit from the intervention), you can consider using the sensory scores light touch total and pin prick total.

Finally, the ASIA Impairment Scale (AIS) ranges from A to E depending on the preservation of motor and sensory functions. Since the participants will be in the sub-acute phase and you propose that the intervention could affect the neural circuits, you could consider changes in the AIS as an outcome.

You will evaluate the SCIM-III as a Demographic variable. The proposed intervention could improve independence, so you can also use it as an outcome measure.

Are you planning to report possible side effects (musculoskeletal pain, hypotension, overuse injuries…)

Did you consider other possible confounders (time since injury, initial AIS, neurologic level, drugs, age)?

Statistical analysis

Which version of the SPSS will you use?

Did you consider an intention-to-treat analysis? Also, please consider subgroup analysis (AIS, Time since injury, neurologic level …)

Discussion

It is not necessary since it is a protocol.

VERSION 1 - AUTHOR RESPONSE

Reviewer: 1
Dr. Jimena Quinzaños-Fresnedo, Instituto Nacional de Rehabilitación
Comments to the Author:
Thank you for the opportunity to read and review this interesting and original article. Its main strength is its potential impact on spinal cord injury health. Additionally, the methodological approach is appropriate, which ensures the project's internal and external validity.

Author Response: We thank Dr Quinzaños-Fresnedo for reviewing our manuscript and highlighting the strength of our study.

However, some concerns must be assessed to improve the project.

1. **Strengths and limitations of the study**
   Since you are presenting a protocol with no results, you cannot affirm that the study "reveals" nor "provides", please consider changing this to "could reveal" or "has the potential to provide".

Author Response: we have revised the strengths and limitations of the study as suggested.

2. **Introduction**
   Consider changing the order of the paragraphs. You start with clinical data, then discuss pathophysiological mechanisms, afterward you return to clinical data, and then again to physiological data. Finally, you present clinical data unrelated to trunk

control. The information provided is important to demonstrate the significance of the work, but it could be better organized to facilitate reading.

Author Response: We have rearranged the paragraphs in the Introduction to improve the overall flow and readability. These changes are highlighted in red in the revised manuscript. We have retained the paragraph describing the exercise and psychological benefits in individuals with SCI in its original position, as this introduces a distinct concept from the physical benefits discussed earlier. We hope the reviewer agrees with our rationale for this structure.

3. **Objective 3: Please consider other clinical, validated data to evaluate improvements (strength, independence, trunk control). Some of them are mentioned later in the manuscript (strength).**

Author Response: Thank you for the recommendation. We have added strength and independence as part of our objective 3 (page 6, line 120-121).

4. **Eligibility criteria: did you consider that subjects with more than 4 months since the injury will complete the intervention in a chronic phase?**

Author Response: We would like to clarify that the intervention will last for 8 weeks. After that, the arm bikes will be returned, and the participants will be kept in the study for another 6 months without undertaking the intervention. With this in mind, all participants, including those with 4-6 months since injury when being recruited, will complete the intervention within 12 months since injury, before moving into the chronic phase.
The 6-month follow-up may occur during the chronic phase of SCI for some participants. We have clarified this in the manuscript (page 10, line 203-205).

5. **Please justify why you will only include incomplete injuries.**

Author Response: The underlying mechanisms of the ACET for trunk control is based on the use of shared neural circuits, such as crossed corticospinal facilitation, between the upper limbs and the trunk[3,4], discovered in individuals with incomplete SCI. Therefore, we will only include people with incomplete SCI in this study. Whether the intervention has the potential to benefit people with complete SCI warrants further investigation.

6. **Exclusion criteria: Did you consider cardiovascular health (previous co-morbidities).**

Author Response: Participants with any contraindication to exercise in an upright posture, based on the evaluation of their medical team, will be excluded from the study. Those with uncontrolled cardiovascular health concerns would likely be excluded from the study. We have specified this in the exclusion criteria (page 6, line 164).

7. **Interventions**
   Please specify the time lapse between the discharge and initiation of the intervention.

Author Response: A typical hospital stay in a SCI rehabilitation unit in the UK is 3 months, although this can vary individually based on the severity of the injury and presence of comorbidities. Hence, we anticipate that participants would complete the intervention before they are discharged from the hospital. If a participant is discharged during the intervention, they will continue the intervention at home. This information is in the TiDieR table (Appendix 4) and we have now clarified this in the main text (page 9, line 198-199).

8. **Outcomes**
   Demographics. Please consider adding previous physical activity and comorbidities.

Author Response: Previous physical activity will be collected during the initial conversation session with participants in the ACET group (please see Appendix 4 TiDieR table for details). Comorbidities will be collected as part of the medical history of the participants; this has now been clarified in the methods (page 10, line 222).

9. **The SCIM-III (and not SCIM-3) is not a questionnaire but a disability scale. A self-reported version exists, the SCIM-SR (Fekete C, Eriks-Hoogland I, Baumberger M, Catz A, Itzkovich M, Lüthi H, Post MWM, von Elm E, Wyss A, Brinkhof MWG. Development and validation of a self-report version of the Spinal Cord Independence Measure (SCIM III). Spinal Cord. 2013 Jan;51(1):40–7).**

Author Response: We have now changed SCIM-3 for SCIM-III and replaced questionnaire with scale throughout the manuscript.

10. **Volitional control of the trunk. Please consider the following paper for the IMUs analysis: Pérez-Sanpablo, A. I., Quinzaños-Fresnedo, J., Romero-Ixtla, M., Aguirre-Güemez, A. V., Rodríguez-Reyes, G., Pérez-Zavala, R., … Quijano-González, Y. (2021). Validation of inertial measurement units for the assessment of trunk control in subjects with spinal cord injury. The Journal of Spinal Cord Medicine, 46(1), 154–163. https://doi.org/10.1080/10790268.2021.1975083.**

Author Response: Thank you for the suggestion. We have now included this reference for the IMUs analysis (page 12, line 248).

11. **Secondary outcomes.**
    The international standards for neurologic classification of spinal cord injury (ISNCSCI) have multiple outcome measures. The motor function is divided into upper extremities motor score (UEMS), and lower extremities motor score (LEMS). Please use the right terms and specify if you will use both or only one.

Author Response: We have now clarified that we will record the upper extremities motor score (UEMS) and lower extremities motor score (LEMS) using the ISNCSCI (page 14, line 300-301).

12. Since trunk control also depends on afferents (and those pathways could benefit from the intervention), you can consider using the sensory scores light touch total and pin prick total.

Author Response: Thank you for the suggestion. We have included that the sensory scores of light touch and pin prick from the INSCSCI will be recorded (page 14; line 297-299).

13. Finally, the ASIA Impairment Scale (AIS) ranges from A to E depending on the preservation of motor and sensory functions. Since the participants will be in the sub-acute phase and you propose that the intervention could affect the neural circuits, you could consider changes in the AIS as an outcome. ASIA B., did they convert into C at discharge/others?

Author Response: Thank you for the suggestion. We have included the AIS as a secondary outcome (page 14; line 297-299).

14. You will evaluate the SCIM-III as a Demographic variable. The proposed intervention could improve independence, so you can also use it as an outcome measure.

Author Response: We have included the SCIM-III as an outcome measure (page 15; line 337-339).

15. Are you planning to report possible side effects (musculoskeletal pain, hypotension, overuse injuries…)

Author Response: Yes, any study-related adverse or serious adverse events will be documented and reported in accordance with the Sponsor's safety reporting procedures (page 18; line 367-369).

16. Did you consider other possible confounders (time since injury, initial AIS, neurologic level, drugs, age)?

Author Response: Possible confounders will be collected as part of the demographics and be included in the statistical analysis where appropriate. We have clarified this in the statistical analysis section (page 18; line 360-361).

17. Statistical analysis
Which version of the SPSS will you use?

Author Response: We have now clarified that we will using the latest version of the SPSS when performing statistical analysis (page 17; line 351-352).

18. Did you consider an intention-to-treat analysis? Also, please consider subgroup analysis (AIS, Time since injury, neurologic level …)

Author Response: Yes, an intention-to-treat analysis will be applied in the statistical analysis (page 17; line396-398). Subgroup analysis (e.g., AIS classification, neurological levels) will be performed if appropriate (page 18, line 356-357).

19. Discussion
It is not necessary since it is a protocol.

Author Response: We included a Discussion section, as this is commonly found in protocols published in *BMJ Open*. We believe it adds value by providing readers with a broader perspective on the protocol and its anticipated contribution. However, we are happy to be guided by the Editor on whether this section should be retained.

References:

1 Braun, V. & Clarke, V. What can "thematic analysis" offer health and wellbeing researchers? *Int J Qual Stud Health Well-being* **9**, 26152 (2014). https://doi.org/10.3402/qhw.v9.26152

2 Mathur, P. *et al.* Supervised and self-directed technology-based dual-task exercise training programme for older adults at risk of falling - Protocol for a feasibility study. *PLoS One* **20**, e0314829 (2025). https://doi.org/10.1371/journal.pone.0314829

3 Chiou, S. Y., Strutton, P. H. & Perez, M. A. Crossed corticospinal facilitation between arm and trunk muscles in humans. *J Neurophysiol* **120**, 2595-2602 (2018). https://doi.org/10.1152/jn.00178.2018

4 Chiou, S. Y., Morris, L., Gou, W., Alexander, E. & Gay, E. Motor cortical circuits contribute to crossed facilitation of trunk muscles induced by rhythmic arm movement. *Sci Rep* **10**, 17067 (2020). https://doi.org/10.1038/s41598-020-74005-z

---

VERSION 2 - REVIEW

**Reviewer**	**1**
**Name**	**Quinzaños-Fresnedo, Jimena**

Affiliation **Instituto Nacional de Rehabilitación, Neurologic Rehabilitation**

Date **30-Jul-2025**

COI

All comments were appropriately attended, the article can be accepted
